# Trends in primary total hip arthroplasty in Spain from 2001 to 2008: Evaluating changes in demographics, comorbidity, incidence rates, length of stay, costs and mortality

**DOI:** 10.1186/1471-2474-12-43

**Published:** 2011-02-09

**Authors:** Rodrigo Jimenez-Garcıa, Manuel Villanueva-Martınez, Cesar Fernandez-de-las-Penas, Valentın Hernandez-Barrera, Antonio Rıos-Luna, Pilar Carrasco Garrido, Ana Lopez de Andres, Isabel Jimenez-Trujillo, Jesus San Roman Montero, Angel Gil-de-Miguel

**Affiliations:** 1 Preventive Medicine and Public Health Teaching and Research Unit, Department of Health Sciences, Universidad Rey Juan Carlos, Madrid, Spain; 2 Orthopedic Department. Hospital Gregorio Marañon. Madrid. Spain; 3 Department of Physical Therapy, Occupational Therapy, Rehabilitation and Physical Medicine, Universidad Rey Juan Carlos, Madrid, Spain; 4 Orthopedic Department. Virgen del Mar Hospital. Almeria. Spain

## Abstract

**Background:**

Hip arthroplasties is one of the most frequent surgical procedures in Spain and are conducted mainly in elderly subjects. We aim to analyze changes in incidence, co-morbidity profile, length of hospital stay (LOHS), costs and in-hospital mortality (IHM) of patients undergoing primary total hip arthroplasty (THA) over an 8-year study period in Spain.

**Methods:**

We selected all surgical admissions in individuals aged ≥40 years who had received a primary THA (ICD-9-CM procedure code 81.51) between 2001 and 2008 from the National Hospital Discharge Database. Age- and sex-specific incidence rates, LOHS, costs and IHM were estimated for each year. Co-morbidity was assessed using the Charlson comorbidity index.

Multivariate analysis of time trends was conducted using Poisson regression. Logistic regression models were conducted to analyze IHM.

**Results:**

We identified a total of 161,791 discharges of patients having undergone THA from 2001 to 2008. Overall crude incidence had increased from 99 to 105 THA per 100.000 inhabitants from 2001 to 2008 (p < 0.001). In 2001, 81% of patients had a Charlson Index of 0, 18.4% of 1-2, and 0.6% > 2 and in 2008, the prevalence of 1-2 or >2 had increased to 20.4% and 1.1% respectively (p < 0.001). The mean LOHS was 13 days in 2001 and decreased to 10.45 days in 2008 (p < 0.001). During the period studied, the mean cost per patient increased from 6,634 to 9,474 Euros. Multivariate analysis shows that from 2001 to 2008 the incidence of THA hospitalizations has significantly increased for both sexes and only men showed a significant reduction in IHM after THA.

**Conclusions:**

The current study provides clear and valid data indicating increased incidence of primary THA in Spain from 2001 to 2008 with concomitant reductions in LOHS, slight reduction IHM, but a significant increase in cost per patient. The health profile of the patient undergoing a THA seems to be worsening in Spain.

## Background

Multiple studies have documented significant qualitative and quantitative improvements in physical function and health related quality of life after total hip arthroplasty (THA) surgery [1-4]. THA surgery is highly successful in relieving pain and has demonstrated to be a cost-effective and durable procedure [1-4]. Recent reports based on National Arthroplasty Registries show that the overall 10-year survival of all THA is over 90% [5,6].

Collecting data about hospitalizations for THA is important at a country level to evaluate the incidence, patient characteristics and outcome of the surgery in variables such as length of stay (LOHS), complications, mortality and burden of disease [7]. Surveys from the United States and other countries have reported a continued growth in the use of THA over the last decades [7-11]. The demand for hip replacement is likely to increase as the result of ageing populations and because the extension of the age range for this treatment [7-11]. Current projections for the United States suggest that from 2005 to 2030 the number of THA will increase by 174%, to nearly 600,000 procedures per year [11].

Liu et al found that the LOHS after a THA has decreased from an average of 8.7 days in 1990-4, to 5.2 days in 1995-9, and to 4.5 days in the most recent time period studied (2000-4). In addition, in-hospital mortality rate has also decreased (0.33% in 1990-4 to 0.29% in 2000-2004) in different countries [7,9,10]. In fact, the economic burden of THA surgery is high, constantly increasing and shows great variation between countries [12,13].

Comparisons of THA rates and outcomes between countries could provide information that would help to understand the possible differences and would also aid for planning the provision of healthcare services [14]. In the absence of a domestic joint replacement registry, the discharge database provides a large alternative information source to describe and analyze the trends and characteristics of THA at a national level [13-15]. Therefore, the aim of this study was to analyze national representative hospital discharge data, collected from 2001 to 2008 years, to elucidate changes in the incidence, comorbidity profiles, LOHS, costs and in-hospital mortality of patients undergoing primary THA in Spain over a 8-year study period.

## Methods

According to the Spanish Health System Organization, each physician must declare, at the time of discharge of each hospitalization all diagnoses and procedures performed, using the code of the International Classification of Disease, 9th revision (ICD-9CM). This information is collected by the Spanish National Hospital Database, namely Conjunto Minimo Basico de Datos (CMBD) that compiles all the public and private hospital data covering hence more than 95% of hospital discharges [16]. The CMBD database includes patients' variables (sex, date of birth), date of admittance, date of discharge, discharge destination (home, decease or other health/social institution), up to 14 discharge diagnosis and up to 20 procedures performed during the admission.

We selected all surgical admissions (elective or emergency admissions) in women and men with 40 years or over, who received a primary THA procedures (ICD-9-CM procedure code 81.51) during 2001-2008 year period. We calculated the yearly age- and sex-specific incidence rates by dividing the number of cases per year per sex and age group, with the corresponding number of person in that population group according to the National Institute of Statistics (INE) reported at December 31 of each year [17]. The incidence rates were expressed in terms of 100,000 inhabitants. The proportion of patients that died during the hospital admission (in-hospital mortality), LOHS, and costs were also estimated for each year studied. Costs were calculated using Diagnosis-Related Groups (DRG) for the disease. DRG represents a medical-economic entity concerning a set of diseases requiring analogous management resources [18]. All costs shown are adjusted for the increment of the inflation in the same period in Spain.

Clinical characteristics included information in overall co-morbidity at the time of surgery, which was assessed by computing the Charlson comorbidity index [19]. The index applies to 19 disease categories that are summated to obtain an overall score for each patient. We divided patients into 3 categories: low index, which corresponds to patients with no previously recorded disease categories in the Charlson comorbidity index; medium index, patients with one or two disease categories; and high index, patients with more than two disease categories.

The primary diagnosis was considered the reason for the THA and was categorized in "osteoarthritis (OA)" and "other reasons".

### Statistical analysis

Quantitative variables are expressed as means, medians, range and inter-quartile range (IQR). Qualitative variables are expressed as frequencies and percentages. Comparisons were done using the chi-square test, Fisher's exact test, Student's t-test, Wilcoxon rank-sum test, ANOVA or Kruskal-Wallis test as appropriate. The multivariate analysis of time trend of study variables were conducted using Poisson and logistic regression models and adjusting by age, sex and other co-variables when appropriate. We then checked for interactions between the independent variables in the regression models. Estimations were made using STATA version 10.1 program (StataCorp LP, Lakeway DriveCollege Station, Texas, USA) and statistical significance was set at two-tailed α < 0 05.

Data were treated with full confidentiality, according to the Spanish legislation. Patient identifiers were deleted before the database was provided to the authors in order to keep strict patient confidentiality. It is not possible to identify patients at individual levels either in this paper or in the database. Given the anonymous and mandatory nature of the data informed consent was not required nor necessary. The Spanish Ministry of Health evaluated the protocol of our investigation and considered that it met all ethical aspects according to the Spanish legislation so provided us the anonymous database.

For all the previous reasons the requirement for ethical approval was not necessary.

## Results

We identified a total of 161,791 discharges of patients (73,248 men and 88,543 women) having undergone THA from 2001 to 2008. Table 1 reflects the total number of procedures performed and the incidence of THA per 100,000 inhabitants in each year. The overall crude incidence has increased from 99 THA to 105 THA per 100.000 inhabitants from 2001 to 2008 (p < 0.001). Among men, incidence significantly increased for all age categories, except among those aged 65-74 years, where it decreased. Among women, increase was found among those aged 75-84 and over 84 years, and decrease was found in the same age group than in men. Overall, only men had a significant increase in the crude incidence over the 8-year study period.

**Table 1:** Age and sex-specific incidence rates for primary THA per 100,000 inhabitants in Spain (2001-8).

		2001	2002	2003	2004	2005	2006	2007	2008	
Age	Sex	N = 18,185	N = 19,029	N = 19,545	N = 19,733	N = 20,461	N = 21,176	N = 21,354	N = 22,308	P*
40-54	Men	29.64	31.90	30.83	31.61	33.97	36.62	37.97	39.14	<0.001
	
	Women	17.54	16.92	16.16	16.80	17.93	17.92	17.30	18.20	0.104

50-64	Men	89.80	99.37	97.41	96.92	103.27	104.48	104.53	110.86	<0.001
	
	Women	74.57	78.92	74.77	75.74	69.90	74.03	75.36	75.29	0.420

65-74	Men	200.39	187.94	197.35	189.43	195.56	190.92	192.18	184.98	0.022
	
	Women	217.76	216.53	220.47	206.90	205.14	206.89	199.46	200.96	<0.001

75-84	Men	164.55	192.34	201.86	201.05	209.11	221.16	208.99	226.10	<0.001
	
	Women	214.47	241.82	242.24	256.66	262.51	265.94	262.04	275.89	<0.001

≥ 85	Men	79.25	73.04	81.41	69.09	65.92	78.75	90.63	101.59	<0.001
	
	Women	100.59	102.27	106.43	109.22	112.70	109.87	120.03	119.35	0.008

Total	Men	94.64	97.99	99.69	97.52	101.55	103.44	102.87	105.71	<0.001
	
	Women	102.80	106.18	105.48	104.78	104.00	104.83	102.88	105.01	0.931
	
	Both	99.05	102.40	102.80	101.42	102.87	104.19	102.88	105.33	<0.001

Incidence rates calculated by dividing the number of cases per year sex and age group with the corresponding number of person in that population group according to the National Institute of Statistics (INE) reported at December 31 each year [17].

*P value for time trend estimated using Poisson regression models

The highest incidence for both sexes was found in the 75-84 years age group. Even more, the highest percent of increase over time was also seen in this group of patients. When we compared incidence by sexes we found higher incidence in women than in men for all age groups over 64 years and higher incidence among men for age groups under 65 years.

Time trends in the prevalence of osteoarthritis, discharge destinations and Charlson comorbidity index are summarized in the Table 2. In 75% of the population undergoing a THA, osteoarthritis was the main reason for surgery. After adjusting by age and sex the prevalence of OA was found to increase over time.

**Table 2:** Prevalence of Osteoarthritis, discharge destinations and Charlson comorbidity index of the THA patients in Spain from 2001 to 2008

		2001	2002	2003	2004	2005	2006	2007	2008	P*
Reason for surgery	Osteoarthritis (%)	74.8	75.8	75.9	76.1	76.3	76.3	75.4	77.0	<0.001
		
	Other (5)	25,2	24,2	24,1	23,9	23,7	23,7	24,6	23	

Discharge destinations	Home (%)	96.8	97.0	96.4	96.4	96.1	95.7	95.9	95.3	<0.001
		
	Health/social institution (%)	3.2	3.0	3.6	3.6	3.9	4.3	4.1	4.7	

Charlson comorbidity index	0 (%)	81.0	81.2	79.9	79.4	79.2	78.7	77.9	78.5	<0.001
		
	1-2 (%)	18.4	18.1	19.3	19.9	19.9	20.4	21.3	20.4	
		
	>2(%)	0.6	0.7	0.8	0.7	0.9	0.9	0.8	1.1	

*P value for time trend estimated using logistic regression adjusted by age and sex.

The proportion of individuals who were discharged to a social or health institution rose significantly from 3.1% in 2001 to 4.6% in 2008. In 2001, 81% of patients had a Charlson Index of 0, 18.4% of 1-2, and 0.6% > 2. In 2008, the proportion of patients who had undergone a THA and had Charlson Index of 1-2 or >2 had increased to 20.4% and 1.1% respectively (p < 0.001).

The mean LOHS for THA admissions was 13 days in 2001, but decreased to 10.45 days in 2008 (p < 0.001). In addition and as expected, it was higher as age increased. A decreasing time trend in the LOHS was seen in all age groups (Table 3).

**Table 3:** Length of stay (LOHS) for primary THA hospitalizations in Spain from 2001 to 2008

		2001	2002	2003	2004	2005	2006	2007	2008	P*
	
Age group		LOHS	LOHS	LOHS	LOHS	LOHS	LOHS	LOHS	LOHS	
40-54*	Mean	11.93	11.15	10.84	10.00	9.70	9.69	8.99	9.06	<0.001
		
	Median	10	9	9	8	8	8	8	8	
		
	IQR	8-13	7-12	7-12	7-11	7-10	6-10	6-9	6-9	

50-64*	Mean	12.02	11.52	11.29	10.53	10.39	10.10	9.61	9.27	<0.001
		
	Median	11	10	9	9	8	8	8	8	
		
	IQR	8-14	8-13	8-13	7-12	7-11	7-11	6-10	6-10	

65-74*	Mean	12.76	12.36	11.75	11.32	10.95	10.67	10.34	10.03	<0.001
		
	Median	11	11	10	9	9	9	9	8	
		
	IQR	9-14	8-14	8-13	8-13	7-12	7-12	7-11	7-11	

75-84	Mean	14.15	13.68	13.31	12.80	12.50	12.31	12.04	11.46	<0.001
		
	Median	12	12	11	11	10	10	9	9	
		
	IQR	9-16	9-15	8-15	8-15	8-14	8-14	8-13	7-13	

≥ 85	Mean	16.20	17.45	16.67	16.27	15.99	15.11	15.37	14.97	0.001
		
	Median	13	14	13	12	13	12	12	11	
		
	IQR	10-18	10-20	10-20	9-18	9-19	9-18	8-18	8-17	

Total	Mean	13.00	12.61	12.18	11.65	11.36	11.13	10.78	10.45	<0.001
		
	Median	11	11	10	10	9	9	9	8	
		
	IQR	9-15	8-14	8-14	8-13	7-13	7-12	7-12	7-11	

IQR Inter quartile range

* P value for comparing means along the study period

The whole hospital costs for THA hospitalizations in Spain were 211 millions of Euros in 2008. During the period studied, the mean cost per patient increased from 6,634 Euros in 2001 to 9,474 Euros in 2008, which represents a 42.8% increment (Table 4). Women had a higher total and mean cost per patient than men in all the years analyzed.

**Table 4:** Hospital costs for primary total hip arthroplasties hospitalizations in Spain from 2001 to 2008

Year	Mean (€)	Median (€)	Percentile 25	Percentile 75	Total cost (Millions of €)	P*
2001	6635	5654	4626	7710	120.65 €	<0.001
	
2002	6731	5889	4283	7495	128.09 €	
	
2003	7418	6145	4916	8603	144.98 €	
	
2004	7654	6309	5232	8502	151.03 €	
	
2005	7345	5890	4581	8404	150.28 €	
	
2006	8754	7107	5528	9476	185.37 €	
	
2007	8625	7234	5626	9645	184.19 €	
	
2008	9474	7297	6385	10034	211.34 €	

* P value for comparing means along the study period

In-hospital mortality (IHM) rate trends according to sex- and age-groups are shown in Table 5. The overall IHM was 0.61% in 2008 (0.55% in women, 0.65% in men) with the highest mortality rates being among men and women aged 85 or over, with figures of 6.1% and 3.6% respectively. Women had lower IHM rates than men in all age groups over 64 years. In fact time trend analysis shows a significant reduction in IHM among men aged 65-74 years, 75-84 years and ≥85 years.

**Table 5:** Incidence of In-Hospital Mortality (IHM) after primary THA in Spain from 2001 to 2008

		2001	2002	2003	2004	2005	2006	2007	2008	
	
		IHM (%)	IHM (%)	IHM (%)	IHM (%)	IHM (%)	IHM (%)	IHM (%)	IHM (%)	p*
40-54	Men	0.00	0.16	0.24	0.15	0.00	0.06	0.17	0.11	0.924
	
	Women	0.15	0.44	0.00	0.70	0.00	0.13	0.00	0.23	0.367

50-64	Men	0.23	0.21	0.46	0.10	0.05	0.13	0.21	0.16	0.239
	
	Women	0.06	0.06	0.06	0.06	0.13	0.12	0.28	0.27	0.018

65-74	Men	0.49	0.49	0.41	0.18	0.39	0.34	0.31	0.19	0.025
	
	Women	0.27	0.47	0.22	0.40	0.26	0.24	0.40	0.30	0.884

75-84	Men	1.68	1.66	1.41	0.80	0.97	1.10	1.26	1.05	0.046
	
	Women	0.77	0.84	0.52	1.06	0.70	0.87	0.69	0.71	0.736

≥ 85	Men	8.75	8.28	3.91	9.55	5.73	5.53	4.90	6.10	0.004
	
	Women	3.85	3.78	5.97	5.63	5.25	3.70	5.45	3.60	0.435

Total	Men	0.75	0.76	0.69	0.47	0.47	0.53	0.60	0.55	0.511
	
	Women	0.54	0.68	0.57	0.87	0.66	0.63	0.78	0.65	0.722
	
	Both	0.63	0.71	0.62	0.69	0.57	0.59	0.69	0.61	0.479

IHD is calculated dividing the number of death by the number of THA hospitalizations in that sex and age group.

*P value for time trend estimated using Poisson regression models

Table 6 and 7 summarizes the results of the multivariate analysis. No interactions between independent variables proved statistically significant. After controlling for possible confounders using Poisson regression models, we observed that the incidence of THA hospitalizations has significantly increased from 2001 to 2008 for men and women (IRR 1.15, 95% CI 1.11-1.18 and 1.07, 95% CI 1.04-1.09 respectively). With regard to IHM, after adjusting the logistic regression model, only men showed a significant reduction in the risk of death after THA from 2001 to 2008 (OR 0.62 CI 95% 0.43-0.90). For both sexes the risk of in hospital death was higher as age and Charlson co-morbidity index increased, and among those who had other than osteoarthritis as the reason for surgery. Finally, in the model including both gender, men had 1.72 times more probabilities of dying during the hospitalization time for a THA than women.

**Table 6:** Multivariate analysis of trends and factors associated with incidence for primary THA

		INCIDENCE OF HOSPITALIZATIONS FOR THA		
		Risk Rate Ratios (95% Confidence Intervals)*		

		**Men**	**Women**	**Both**

Age	40-54	-	-	-
	
	55-64	2.96(2.89-3.03)	4.30 (4.17-4.43)	3.40 (3.34-3.46)
	
	65-74	5.65 (5.52-5.77)	12.04 (11.71-12.38)	7.84 (7.71-7.97)
	
	75-84	5.98 (5.84-6.12)	14.57 (14.17-14.98)	9.09 (8.93-9.25)
	
	≥85	2.37 (2.25-2.50)	6.37 (6.14-6.21)	3.97 (3.86-4.08)

Sex	Women	NA	NA	-
	
	Men	NA	NA	1.03 (1.02-1.04)

Reason for surgery	OA	-	-	-
	
	Other	0,21 (0,20-0,22)	0,42 (0,41-0,43)	0,32 (0,31-0,33)

Charlson comorbidity index	0	-	-	-
	
	1-2	0.24 (0.24-0.25)	0.25 (0.25-0.26)	0.25 (0.24-0.25)
	
	>2	0.012 (0.011-0.013)	0.008 (0.008-0.009)	0.010 (0.009-0.011)

YEAR	2001	-	-	-
	
	2002	1.04 (1.00-1.07)	1.04 (1.01-1.07)	1.04 (1.02-1.06)
	
	2003	1.06 (1.03-1.09)	1.04 (1.01-1.07)	1.05 (1.03-1.07)
	
	2004	1.04 (1.01-1.07)	1.04 (1.01-1.07)	1.04 (1.02-1.06)
	
	2005	1.09 (1.06-1.12)	1.04 (1.01-1.07)	1.06 (1.04-1.08)
	
	2006	1.11 (1.08-1.15)	1.05 (1.02-1.08)	1.08 (1.06-1.10)
	
	2007	1.11 (1.08-1.15)	1.04 (1.01-1.07)	1.07 (1.05-1.09)
	
	2008	1.15 (1.11-1.18)	1.07 (1.04-1.09)	1.10 (1.08-1.12)

Calculated using multivariate Poisson regression Dependent variable "Incidence of hospitalizations for THA".

The independent variables included in the models are those shown in the table.

OA Osteoarthritis

**Table 7:** Multivariate analysis of trends and factors associated with in-hospital deaths after primary THA

		IN-HOSPITAL DEATHS AFTER HOSPITALIZATIONS FOR THA		
		**Odds Ratios (95% Confidence Intervals**		

		**Men**	**Women**	**Both**

Age	40-54	-	-	-
	
	55-64	2.16 (1.13-4.14)	0.76 (0.37-1.59)	1.49 (0.92-2.41)
	
	65-74	4.29 (2.39-7.71)	1.61 (0.88-2.93)	3.10 (2.03-4.71)
	
	75-84	11.81 (6.70-20.82)	2.95 (1.65-5.29)	6.81 (4.52-10.25)
	
	≥85	28.25 (15.67-50.91)	10.45 (5.81-18.80)	20.83 (13.74-31.58)

Sex	Women	NA	NA	-
	
	Men	NA	NA	1.72 (1.50-2.01)

Reason for surgery	OA	-	-	-
	
	Other	6,67 (5,26-8,33)	5,56 (4,35-6,67)	5,88 (5,00-7,14)

Charlson comorbidity index	0	-	-	-
	
	1-2	4.40 (3.54-5.47)	4.38 (3.66-5.24)	4.39 (3.82-5.04)
	
	>2	18.20 (12.93-25.63)	17.78 (12.78-24.73)	18.08 (14.28-22.89)

YEAR	2001	-	-	-
	
	2002	0.97 (0.67-1.40)	1.30 (0.91-1.86)	1.13 (0.88-1.46)
	
	2003	0.81 (0.56-1.17)	0.99 (0.69-1.45)	0.91 (0.70-1.18)
	
	2004	0.57 (0.38-0.86)	1.56 (1.11-2.20)	1.04 (0.81-1.34)
	
	2005	0.53 (0.36-0.80)	1.13 (0.79-1.62)	0.82 (0.63-1.07)
	
	2006	0.57 (0.39-0.84)	1.07 (0.75-1.54)	0.81 (0.62-1.05)
	
	2007	0.59 (0.41-0.86)	1.20 (0.85-1.70)	0.88 (0.68-1.13)
	
	2008	0.54 (0.37-0.79)	1.04 (0.73-1.48)	0.78 (0.60-1.00)

Calculate using logistic regression. Dependent variable "In hospital death after hospitalizations for THA"

The independent variables included in the models are those shown in the table. OA Osteoarthritis

## Discussion

In this observational study involving 161,791 THA, we found a significant increase in the incidence of this surgical procedure from 2001 to 2008 in the Spanish population. When reporting on incidences of THA, it is necessary to make comparisons with other countries. Lohmander et al estimated, among people suffering OA, the crude incidence of primary THA (rate per year per 100,000 persons) obtaining results between 70 for Iceland and 99 for Sweden in 2000 [14]. In Australia, Wells et al obtained an incidence of 60.9 per 100,000 [20]. Our estimated incidence for patients with OA was 74 per 100,000 in 2001 which falls within previously ranges. However, comparison between studies is too complicated due to country differences related to population demographic structure (age, sex, race), timing of the studies, and methods of reporting and should be interpreted with caution [20].

The present results agree with those studies conducted in other countries where females have also significantly higher incidence than males [5,7,14,21,22]. In Spain, females constituted 55% of the patients over the entire period. In the US, for the period 2000-4 females accounted for 57% of discharges with diagnosis of total hip arthroplasty [7]. Analysis of the arthroplasty registries of Sweden, Denmark, and Norway showed that females represented 58% of the patients in Denmark, 60% in Sweden, and 70% in Norway [5]. In addition, female patients had higher incidence rates of THA than men in all age groups [5]. In Japan, only 16.1% of THA were conducted in men during 2006-7 period [21]. Previous research suggests that OA is more prevalent and incapacitating in females than in males [5,14,22]. Differences in referral practices and patient willingness or other unknown factors may explain discrepancies in THA implantation between gender and differences between countries in sex-specific incidence [5,14,22].

We also found that men incidence increased significantly for all age categories except among those aged 65-74, where decreased. In women, the increase was found among those aged 75 years and over. In the US, from 1990 to 2004, the use of THA increased for most age categories, but unlike the current study, a decrease in the group of patients over 84 years was seen [7]. We believe that the increase in older age groups in Spain may be a consequence of concurrent advances during this same time period in surgical techniques, implant materials and designs and peri-operative care, that has allowed the extension of surgical indications to older and sicker patients [9,10,23]. A study conducted in The Netherlands and Sweden found that only 3% and 15%, respectively, of increase in THA incidence may be explained by changes in the population structure by age [23]. In fact, the results of the Poisson regression analysis confirm that the increment in incidence of THA in men and also in women became significant after adjusting for potential confounders. Other authors using multivariate models have reached the same conclusions, verifying therefore that the increase in incidence of THA is not only a consequence of the population growth or ageing [7,9-11].

Primary osteoarthritis, as the most frequent diagnosis among THA patients is constantly found in Europe and elsewhere [5,13,14,21]. Havelin et al showed prevalence of OA of 77.6% in Denmark, 78.8% in Sweden and 73.7% in Norway [5] values almost identical to those reported in the current study for Spain. In Japan, 80.6% of the patients who received THA during 2006-7 have been diagnosed of OA [21].

In Spain, the number of high-risk surgical patients has increased over last 8 years as shown by the analysis of Charlson comorbidity index. This same trend has been previously described in other studies [7,9,10,21]. Aging of population will undoubtedly make patient management after THA more complex in the future [7,9,10,21]. Even more, this increase in co-morbidities in THA patients after adjusting by age, reflect the declining health status of the general Spanish population as previously suggested [24-26].

The LOHS decreased signficantly from an average of 13 days in 2001 to 10.45 days in 2008 in Spain. The mean nationwide LOHS for Spain is longer than that described in other countries such as Denmark, USA or Taiwan, but shorter than in Japan [7,9,21,27,28]. Reasons for the decrease in the LOHS overtime suggested by other authors that may be well applies to Spain include: more specialized departments with multidisciplinary and multimodal teams using more efficient means to rehabilitate and discharge patients and, as found in our study, the increased rate of discharges to short and long-term care facilities [7,13,27,28]. The trend of hospital discharges to medium-care institutions may reflect the indiosincratic problem of a Public Health System. Neither patients accept nor doctors promote a complete discharge until the patient has recovered a certain degree of autonomy as they are not directly penalty by the cost of the procedure.

In all the years analyzed most THA admissions were elective (82-84%) with the remaining 16-18% through the emergency room. The changes in these proportions showed no significant time trend.

In Spain the total cost of primary THA has increased from 120.65 millions of Euros in 2001 to 211.34 millions in year 2008, this represents a 75% increase. The average cost per patient has risen from 6,634 Euros in 2001 to 9,473 Euros in 2008 (42.8% increment). In France whole hospital cost for primary joint replacement for coxarthrosis (N = 69,948) was 590.6 millions of Euros in 2004, with a mean cost per patient of 8,318 Euros [13]. A recent study comparing the cost of primary hip replacement within 9 member states of the European Union found that the total cost of treatment per patient ranged from 1,290 Euros (Hungary) to 8,739 Euros (The Netherlands) [12]. The charge in the USA has been found to be higher [29]. In any case, and as previously commented, comparison of LOHS and costs between countries is too difficult because differences in demographics and specific healthcare system organization and financing [12,13].

A striking result of our study is that, despite the reduction in LOHS, the cost has increased. We believe that this finding may be associated with greater incidence of risk factors and comorbidities in THA patients, higher cost of surgical materials and postoperative care, or an increase in cost of fee-for-service [10,29]. Furthermore, the fact that in Spain the payment system to hospitals has been increasingly associated with the complexity of the patients treated, may have led hospitals to prioritize the codification of those diseases that are associated with increases in the LOHS and in-hospital mortality [10].

The overall in-hospital mortality (IHM) after primary THA in Spain ranged from 0.71% to 0.59% over 2001-08. These findings are higher than those reported in other countries [7,21,27]. Doro et al analyzed 275,813 primary THA for OA from 1988 to 2000 using the US Nationwide Inpatient Sample database and observed that in-hospital mortality was 0.16% in the highest volume hospitals and 0.29% in the lowest volume [27]. More recently, and using the same database, Liu et al have described an in-hospital mortality rate of 0.29% in the 2000-2004 period (n = 953,130) for all primary THA beside the diagnosis [7]. In Japan, a report including 13, 537 THA conducted in 2006-7, found an IHM of 0.23% [21]. Allepuz A et al, using a method similar to ours described almost identical IHM for Catalonia (Spain) in the period 200-2005 [10].

Considering that comparisons are not possible due to the differences in patients and system characteristics, the results of the present study call for urgent research assessing the reasons for such high mortality in Spain. The large number of surgeries conduced in the oldest patients (≥85 years) that has increased sharply in last years is surely contributing to this high IHM. Future studies are needed to confirm this assumption.

Results from the regression analysis show that over the entire period, men exhibited 72% more probability of dying as a consequence of THA than women. Further, the risk of death also increased with age, number of commorbid conditions and having other than OA as reason for THA. These finding are consistent with previous studies showing that the most important risk factors for excessive early postoperative mortality for THA are male gender, older age and co-morbidity [7,15,27].

Finally, we found that increased incidence of THAs was coupled with a slightly reduced rate of in-hospital mortality among men, which is similar to previous reports [7]. Beside those factors previoully commented expaining the increased incidence of THA and the reduction in the LOHS, we agree with others that the increased volume and the use of THAs in-itself has possibly helped to reduce mortality [7,27,30,31].

We should recognize strengths and limitations of the current study. The main strength of the current study lies in a large sample size and standardized methodology maintained constant along the study period. Futhremore, discharge databases have been widely used by other authors to assess outcomes, burden and trends of THA [7,10,13,15,21,27]. Nevertheless, our study presents some limitations. Firstly, a potential source of bias comes from relying on administrative registries as several discrepancies between administrative data and audited and validated clinical data have been suggested [32-35]. However, others have reported that this information was fairly reliable [32-35]. Even more, coding may have improved over the study period resulting in higher comorbidities [10]. Secondly, outcomes were limited to the variables coded, which do not include relevant data such as type of prosthesis fixation, pharmacologic treatments or other factors such as function and pain relief, among others. Therefore, outcomes as LOHS and discharge destination may have been influenced by other covariates different from postoperative complications. In such scenario, only in-hospital mortality can be used to draw direct conclusions on the complication rate in the curren study [27]. Thirdly, we only focused on hospital admissions in people aged over 40 years. Indeed, hospitalizations in people aged under 40 years represented < 2% of all hospitalizations for THA in Spain and other countries [5,10,13]. Lastly, the data in the CMBD is anonymous, with the result that it is impossible to trace patients who underwent a contralateral procedure during the same period. Despite these limitations, the CMBD discharge database has the advantage of being mandated by the National Public Health System and it includes almost 100% of admissions in Spain[16]. In addition, Spain is a large country with a public heatlh system providing full free of charge medical and surgical services to the entire population, so patients came from a variety of socioeconomic categories improving the external validity of the current results.

In our opinion, the best option to obtain more extense, reliable and accuarate information would be to create a national arthroplasty register like is already available in other European countries and Australia [5,14,20]. Implating registries are also necessarry as a surveillence system that would allow tracing new models and early idenfication and withdrow of those with worse results than previous models. In the absence of a formal domestic registry, the incidence, IHM rates and cost estimation reported in this study provide the best possible available information.

## Conclusions

The current study provides clear and valid data indicating an increase in incidence of primary THA in Spain from 2001 to 2008 with concomitant reductions in LOHS and slight reduction in-hospital mortality, but a significant increase in cost per patient. The health profile of the patient undergoing a THA seems to be worsening in Spain. This time trend may be useful for planning future resources, e.g. surgical and rehabilitation centers or specialist consultations.

## List of Abbreviations

IHM: In-hospital mortality; LOHS: length of hospital stay; THA: Total hip arthroplasty; CMBD: Spanish National Hospital Database, namely Conjunto Minimo Basico de Datos

## Competing interests

The authors declare that they have no competing interests.

## Authors' contributions

RJG Conception and design, acquisition of data, analysis and interpretation of data and drafting the manuscript. MVM Conception and design, acquisition of data, interpretation of data and drafting the manuscript. CFDP Conception and design, interpretation of data and drafting the manuscript. VHB. Acquisition of data, analysis and interpretation of data and drafting the manuscript. ARL. Analysis and Interpretation of data and drafting the manuscript. PCG Analysis and Interpretation of data and drafting the manuscript. ALDA Analysis and Interpretation of data and drafting the manuscript. JSRM Analysis and Interpretation of data and drafting the manuscript. AGDM Conception and design and revising the manuscript critically for important intellectual content;

All authors have given final approval of the version to be published.

## Pre-publication history

The pre-publication history for this paper can be accessed here:

http://www.biomedcentral.com/1471-2474/12/43/prepub
